# Bitter taste receptor agonists regulate epithelial two-pore potassium channels via cAMP signaling

**DOI:** 10.1186/s12931-021-01631-0

**Published:** 2021-01-28

**Authors:** Michael A. Kohanski, Lauren Brown, Melissa Orr, Li Hui Tan, Nithin D. Adappa, James N. Palmer, Ronald C. Rubenstein, Noam A. Cohen

**Affiliations:** 1 Department of Otorhinolaryngology-Head and Neck Surgery, Division of Rhinology, University of Pennsylvania Medical Center, Perelman School of Medicine, 5th Floor Ravdin Building, 3400 Spruce Street, Philadelphia, PA USA; 2 Cystic Fibrosis Center, The Children’s Hospital of Philadelphia, Philadelphia, PA USA; 3 Department of Pediatrics, University of Pennsylvania, Perelman School of Medicine, Philadelphia, PA USA; 4 Division of Allergy and Pulmonary Medicine, Department of Pediatrics, Washington University in St. Louis School of Medicine, St. Louis, MO USA; 5 Corporal Michael J. Crescenz Veterans Administration Medical Center, Philadelphia, PA USA; 6 Monell Chemical Senses Institute, Philadelphia, PA USA

**Keywords:** Denatonium, Tuft cell, SCC, Antimicrobial peptide, Defensin, Chronic rhinosinusitis, K2P, Potassium channel

## Abstract

**Background:**

Epithelial solitary chemosensory cell (tuft cell) bitter taste signal transduction occurs through G protein coupled receptors and calcium-dependent signaling pathways. Type II taste cells, which utilize the same bitter taste signal transduction pathways, may also utilize cyclic adenosine monophosphate (cAMP) as an independent signaling messenger in addition to calcium.

**Methods:**

In this work we utilized specific pharmacologic inhibitors to interrogate the short circuit current (Isc) of polarized nasal epithelial cells mounted in Ussing chambers to assess the electrophysiologic changes associated with bitter agonist (denatonium) treatment. We also assessed release of human β-defensin-2 from polarized nasal epithelial cultures following treatment with denatonium benzoate and/or potassium channel inhibitors.

**Results:**

We demonstrate that the bitter taste receptor agonist, denatonium, decreases human respiratory epithelial two-pore potassium (K2P) current in polarized nasal epithelial cells mounted in Ussing chambers. Our data further suggest that this occurs via a cAMP-dependent signaling pathway. We also demonstrate that this decrease in potassium current lowers the threshold for denatonium to stimulate human β-defensin-2 release.

**Conclusions:**

These data thus demonstrate that, in addition to taste transducing calcium-dependent signaling, bitter taste receptor agonists can also activate cAMP-dependent respiratory epithelial signaling pathways to modulate K2P currents. Bitter-agonist regulation of potassium currents may therefore serve as a means of rapid regional epithelial signaling, and further study of these pathways may provide new insights into regulation of mucosal ionic composition and innate mechanisms of epithelial defense.

## Background

Epithelial solitary chemosensory cells (SCCs), also known as tuft cells, detect a wide range of pathogens, metabolites, and bitter compounds [1–9] and these specialized cells have both taste and inflammatory functions [8, 10, 11]. SCCs express proteins associated with bitter taste pathways including transient receptor cation channel subfamily M member 5 (TRPM5), the gustatory G_α_-protein, gustducin (GNAT3), phospholipase Cβ2 (PLCβ2), and an array of bitter and sweet taste receptors [4, 12]. In the human upper airway, bitter taste receptors (T2Rs, ~ 25 encoded in the human genome) are expressed on both SCCs and ciliated epithelial cells, with apparent mutually exclusive expression of specific T2Rs for each cell type. In contrast, gustducin expression is only observed in SCCs [4, 13].

Early work on bitter taste G Protein-Coupled Receptors (GPCRs) focused on the relative contributions of G_α_ vs G_βγ_-mediated pathways in taste cells. Gustatory taste signaling in type II taste cells, which are most similar in terms of taste transduction pathways to SCCs, occurs through G_βγ_-mediated signaling involving PLCβ2, generation of inositol triphosphate (IP_3_) and subsequent TRPM5-dependent calcium-mediated paracrine signaling involving release of adenosine triphosphate (ATP) and acetylcholine [14–17], while G_α_-gustducin activates phosphodiesterases to decrease cyclic adenosine monophosphate (cAMP) [14]. In taste cells, low levels of cAMP may be necessary for robust calcium signaling to occur [18], and cAMP-mediated signaling may also be independent of calcium as a downstream messenger of taste receptors [19].

Studies of SCC-mediated epithelial signal transduction have predominantly focused on the phenotypes associated with the TRPM5-dependent calcium signaling pathways. In mouse respiratory mucosa, bitter agonist stimulation of SCCs triggers a gustducin- and TRPM5-dependent increase in intracellular calcium in the epithelial cells that triggers a trigeminal-apnea response [8]. Stimulation of human sinonasal SCCs with the bitter tasting molecule denatonium, which broadly targets gustducin-coupled T2Rs expressed on SCCs [8, 20] and does not stimulate downstream effects of nasal ciliary T2Rs [21], results in a localized calcium flux in the epithelium and release of β-defensins through a PLCβ-2 and TRPM5 dependent process to contribute to the innate immune response [4]. Bitter taste receptor agonists can also decrease epithelial cAMP levels [22, 23], however, whether or not G_α_-gustducin activates phosphodiesterases in SCCs as the mechanism of this effect is largely unknown.

Here we test polarized nasal epithelial monolayers in Ussing chambers to further interrogate the human respiratory epithelial response to the bitter agonist denatonium, and to specifically test the hypothesis that bitter taste receptor mediated changes in epithelial ion currents relies on cAMP. Our data demonstrate that denatonium benzoate treatment leads to inhibition of human respiratory epithelial two-pore potassium (K2P) channel currents, and further suggest that this occurs via a cAMP-dependent pathway. We also demonstrate that closure of epithelial potassium leak channels lowers the threshold for denatonium-mediated human β-defensin-2 (HBD-2). This work thus demonstrates that, in addition to taste transducing calcium-dependent signaling, bitter taste receptor agonists can also modulate epithelial signaling pathways that may result in enhancement of host defense.

## Methods

### Reagents and experimental solutions

Materials for preforming cell culture were obtained as previously described [4]. Bupivacaine, clotrimazole, and lactisole were obtained from Cayman Chemical (Ann Arbor, MI, USA). Clofilium, forskolin, amiloride, IBMX (3-isobutyl-1-methylxanthine), pertussis toxin, CFTR_inh_-172 (inh172) and denatonium benzoate were from Millipore Sigma (St Louis, MO, USA).

### Tissue acquisition

Residual tissue and/or secretions were obtained from patients recruited from the Department of Otorhinolaryngology – Head and Neck Surgery, Division of Rhinology, University of Pennsylvania, with informed consent; this protocol was approved by the Institutional Review Board (protocol #800614). Patients undergoing sinonasal surgery for chronic rhinosinusitis (CRS) met the inclusion criteria for recruitment. Diagnosis of CRS or chronic rhinosinusitis with nasal polyps (CRSwNP) was per established clinical diagnostic standards [24, 25]. Exclusion criteria included a history of systemic diseases, especially those with characteristic sinonasal symptomatology (e.g., granulomatosis polyangitis, sarcoidosis, cystic fibrosis, immunodeficiency). Tissue for establishing air–liquid interface (ALI) cultures was taken from nasal polyps in the ethmoid sinus.

### Human sinonasal ALI culture

Sinonasal mucosal specimens were acquired from residual clinical material obtained during sinonasal surgery as described above. ALI cultures were established from enzymatically dissociated human sinonasal epithelial cells (HSECs), as previously described [4, 26, 27], and grown to confluence with proliferation medium consisting of bronchial epithelial basal medium [BEBM (Lonza; Basel, Switzerland)] supplemented with Lonza epithelial cell growth medium singlequots, 100 U/ml penicillin and 100 µg /ml streptomycin for 7 days. Cells were then dissociated with trypsin for 2 min at 37^ °^C. Trypsinization was quenched using media containing 2% fetal bovine serum and the cell pellet was washed three times in fresh proliferation media and subsequently seeded on porous polyester membranes (about 6 × 10^4^ cells per membrane) in cell culture inserts [Transwell-clear, 12-mm diameter, 0.4 µm pores; Corning (Corning, NY, USA)]. After achieving confluence, as determined by inspection of transwells via light microscopy, the culture medium was removed from the upper compartment and the epithelium was allowed to differentiate under air–liquid interface (ALI) conditions for at least 3 weeks by using the differentiation medium (consisting of 1:1 Dulbecco’s Modified Eagle Medium [DMEM, (Thermo Fisher Scientific, Waltham, MA, USA)] and BEBM (Lonza), with Lonza epithelial cell growth medium singlequots, supplemented with 100 UI/ml penicillin, 100 µg/ml streptomycin, 0.1 nM retinoic acid (Millipore Sigma), and 2% Nu-Serum (Corning) in the basal compartment. For experiments involving pertussis toxin, pertussis toxin was dissolved in 1X Hank’s balanced salt solution (HBSS) (no glucose, + HEPES (4-(2-hydroxyethyl)-1-piperazineethanesulfonic acid), pH 7.4). ALI cultures were pretreated for 16-h with pertussis toxin (100 ng/ml) added to the media in the basal chamber at 37 °C, 5%CO2 prior to experimentation.

### Ussing chamber experiments

Differentiated ALI cultures treated as described, and when transepithelial resistance was > 300 Ω cm^2^ as assessed by a volt-ohm meter (World Precision Instruments, Sarasota, FL, USA) were mounted in vertical Ussing chambers (Physiologic Instruments, San Diego, CA, USA) and assessed under short circuit conditions. Symmetric solutions of 1X phosphate buffered saline (PBS) pH 7.4 containing 10 mM glucose and 5 mM lactisole (to block sweet taste receptors) was used in the apical and basal chambers. All experiments were performed at 37 °C. Transepithelial resistance was monitored by ± 2 mV bidirectional pulses every 20 s and calculated by Ohm’s Law. For the presentation of these experimental data, we define a positive Isc (short circuit current) as a net positive ion movement from the basolateral to the apical/mucosal surface. All experiments were performed in at least duplicates with primary cells.

Denatonium benzoate solutions were made in 1X PBS containing 10 mM glucose, 5 mM lactisole and pH was adjusted to 7.4. This PBS solution served as the vehicle control for denatonium benzoate unless otherwise indicated. Compounds used for pharmacologic interrogation of epithelial cultures were dissolved in dimethyl sulphoxide (DMSO) and used at the following concentrations: bupivacaine (0.2 mM), clotrimazole (30 µM), clofilium (100 µM), forskolin (10 µM), amiloride (10 µM), IBMX (100 µM), CFTR_inh_-172 (inh172) (10 µM). Where indicated in the results (vehicle), an equivalent volume of DMSO was added to cultures as a vehicle control.

### Antimicrobial peptide release assays

The apical surface of mature ALI cultures were washed 3 times with 1X HBSS (no glucose, + HEPES, pH 7.4) and basal chamber media was changed 24 h prior to assessment of antimicrobial peptide release. On the day of the experiment, cultures were rinsed two times with 1X HBSS. To quantify anti-microbial peptide release, vehicle (DMSO) or apical potassium channel blocker (0.2 mM bupivacaine) was applied to the apical surface of the ALI culture in 25 µL of 1X HBSS. Fresh media containing the respective concentration of vehicle (DMSO) or the indicated basal potassium channel blockers (30 µM clofilium, 100 µM clotrimazole) was placed in the basal chamber and cultures were incubated for 15 min at 37 °C, 5% CO2. Cultures were then treated apically with an additional 25 µL HBSS solution (maintaining the concentration of DMSO or bupivacaine) with or without denatonium benzoate (final concentration 1 mM) and incubated for an additional 15 min at 37 °C, 5% CO2. Apical fluid was collected, centrifuged for 5 min and then frozen at − 80 °C for anti-microbial peptide (defensin) quantification. All experiments were performed using cultures from a minimum of 3 unique patient acquired samples.

### HBD-2 ELISA

Human beta-defensin-2 was quantified in samples of apical fluid by ELISA (LSBio, Seattle, WA, USA) per manufacturer’s instruction.

### Data analysis

Data were analyzed using Prism (Graphpad, San Diego, CA, USA) For experiments comparing 2 groups, significance was determined by Student’s t-test, or the non-parametric Mann–Whitney U tests if the data were not normally distributed. For analysis of HBD-2 ELISA results, vehicle samples below the limit of detection were entered as 0. Statistical significance was determined by non-parametric analysis using the Kruskal–Wallis test followed by Dunn’s multiple comparison with statistical significance *p* ≤ 0.05. For all other comparisons across multiple groups, analysis of variance (ANOVA) was performed. If a significant (*p* ≤ 0.05) difference among means was found, then pairwise post hoc Tukey’s multiple comparisons testing was performed to assess for significant differences between each group. A *p* value of ≤ 0.05 was taken as indicating statistical significance.

## Results

### Denatonium benzoate treatment increases the short circuit current (Isc) and transepithelial resistance of human sinonasal epithelial cells.

**Fig. 1:**
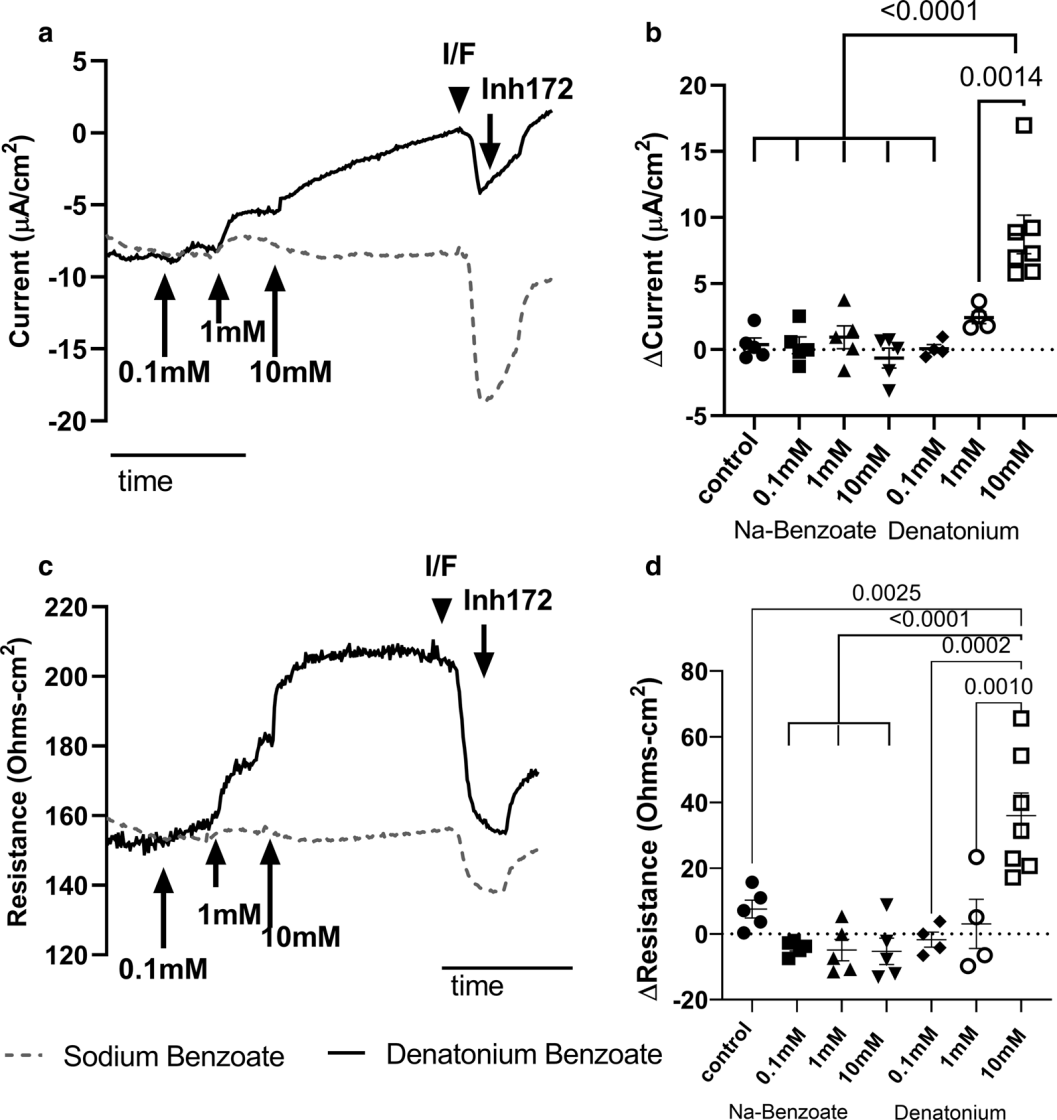
Denatonium benzoate treatment increases the short circuit current (Isc) and transepithelial resistance of human sinonasal epithelial cells. Ussing chamber experiments examining Isc **a**, representative tracing; **b** summary data) and transepithelial resistance (Panel C, representative tracing, Panel D summary data) following treatment with increasing concentrations of denatonium benzoate (solid line) or sodium benzoate (dashed line). Characteristically greater negative Isc with subsequent treatment with 100 µM IBMX/10 µM Forskolin (I/F) that is blocked by 10 µM CFTR_inh_-172 treatment suggests the expected presence of CFTR in these cells. There is a significant increase in the Isc **b** (*p* < 0.0005) and transepithelial resistance **d** (*p* < 0.005) following treatment with increasing concentrations of denatonium benzoate but not with sodium benzoate. Shown in **b** and **d** are the individual data points, mean and standard error of the mean

To explore the electrophysiologic changes in sinonasal epithelia associated with denatonium benzoate treatment, we utilized Ussing chambers to interrogate the short circuit current (Isc) and transepithelial resistance of human sinonasal cells grown under ALI conditions. These cultures were exposed to increasing concentrations of denatonium benzoate on the apical surface (Fig. 1). Increasing concentrations of denatonium benzoate resulted in a positive change in Isc (Fig. 1a, b) and increased transepithelial resistance in these respiratory epithelial cultures (Fig. 1c, d). These observed increases in Isc and transepithelial resistance were only significant at 10 mM denatonium benzoate with a trend toward increased change in Isc also seen at 1 mM denatonium benzoate (Fig. 1b, d). The observed positive change in Isc reflects increased net movement of positive charge towards the apical/mucosal surface of the epithelium, either from net cation secretion (defined as serosal to mucosal transport) or net anion absorption (defined as serosal to mucosal transport). The observed current and resistance changes are specific to denatonium and not related to the associated benzoate component of denatonium benzoate, as no increase in current or resistance was observed following treatment with equimolar sodium benzoate (Fig. 1).

### Denatonium-induced current changes are reversible and cAMP-dependent

**Fig. 2:**
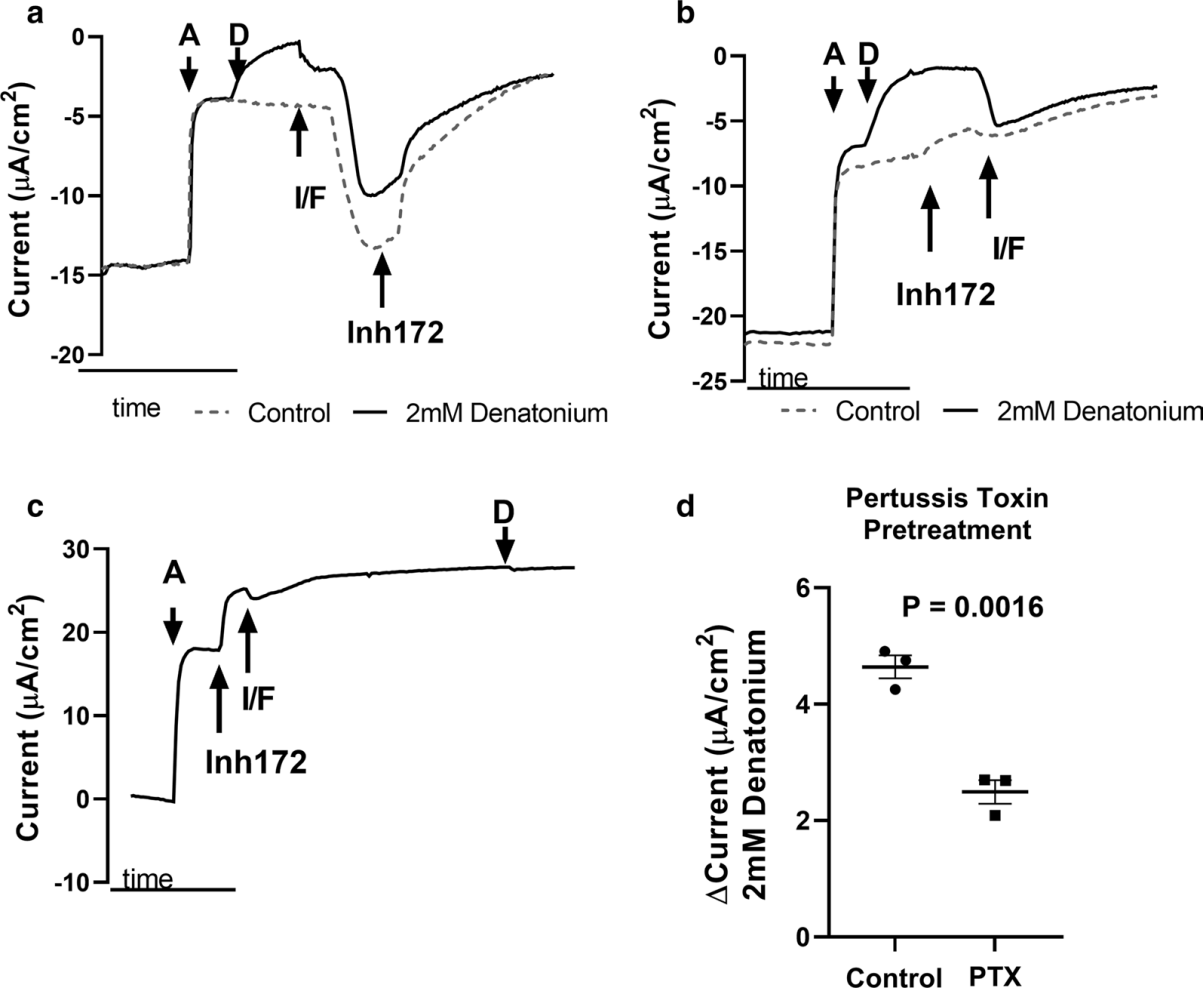
Denatonium benzoate-induced current changes are reversible by cAMP treatment. Representative Ussing chamber tracings of human sinonasal ALI cultures treated with 10 µM amiloride **a** followed by vehicle (dashed line) or 2 mM denatonium benzoate (solid line) and then **a** 100 µM IBMX/10 µM Forskolin (I/F) followed by 10 µM CFTR_inh_-172 (inh172) or **b** inh172 followed by I/F. These data demonstrate denatonium-induced current changes are reversible with an increase in cAMP (I/F treatment) even in the presence of inh172 and are therefore independent of CFTR function. **c** Representative tracing demonstrating that pretreatment with both inh172 and I/F to blocks denatonium-mediated current changes. **d** 100 ng/ml pertussis toxin pretreatment decreases denatonium (2 mM)-mediated change in Isc. Shown in **d** are the individual data points, mean and standard error of the mean

The influence of certain epithelial ion channels on these denatonium-mediated changes in Isc was assessed using (i.e. amiloride (10 µM) for ENaC, CFTR_inh_-172 (10 µM) for CFTR, IBMX (100 µM) and Forskolin (10 µM) for increasing cAMP concentrations). We tested whether the changes in Isc observed with denatonium benzoate treatment are influenced by changes in cAMP through order of addition experiments. We found that denatonium benzoate-mediated current changes were reversed by treatment with 100 µM IBMX and 10 µM Forskolin (increase intracellular cAMP levels), and that these current changes were at least partially due to activation of CFTR, as subsequent treatment with CFTR_inh-_172 decreased the magnitude of these currents towards pre-IBMX/Forskolin levels (Fig. 2a). To determine if the reversal of the denatonium current is due to changes in cAMP or is wholly CFTR-related, we blocked CFTR in denatonium benzoate-treated cultures prior to stimulation of cAMP accumulation with IBMX/forskolin, and nevertheless still observed a reversal of the denatonium-stimulated increased in Isc (Fig. 2b). These data suggest that the denatonium-mediated changes in Isc are reversible by increasing intracellular cAMP, and that this occurs through an ion channel independent of CFTR (Fig. 2b). In fact, increasing intracellular cAMP levels by pre-treatment with 100 µM IBMX/10 µM forskolin blocked denatonium-induced changes in Isc when CFTR-mediated Isc was also inhibited (Fig. 2c). We then pre-treated sinonasal ALI cultures with or without pertussis toxin for 16-h which inactivates G_gustducin_ [28] as well as G_α(i/o)_ and can also increase intracellular cAMP by decreasing the inhibition of adenylyl cyclase by inhibitory G proteins [29, 30]. We found that 100 ng/ml pertussis toxin pretreatment significantly decreases denatonium-mediated change in Isc (Fig. 2d) providing additional evidence that denatonium-mediated changes in epithelial current are likely regulated or reversed by cAMP.

### Denatonium-induced current changes occur through closure of potassium channels

We next sought to further identify the relevant ion(s) and ion channel(s) associated with the change in epithelial Isc observed with denatonium benzoate treatment. These denatonium-induced Isc changes are likely not mediated by ENaC, as amiloride pre-treatment of these ALI cultures does not block denatonium-mediated current changes (Fig. 2). Furthermore, blockade of CFTR does not alter these currents (Fig. 2b), suggesting that these denatonium-induced currents are not determined by chloride fluxes through CFTR.

**Fig. 3:**
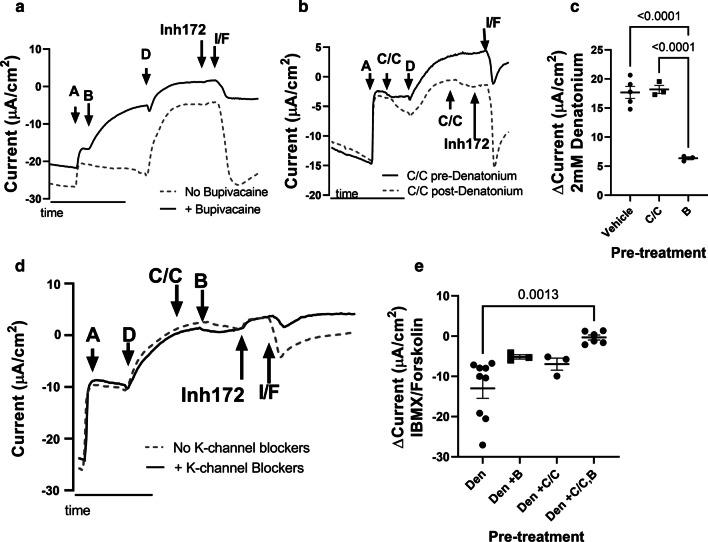
Denatonium benzoate-induced current changes occur through K2P channels. Representative Ussing chamber current tracings of human sinonasal ALI cultures treated with 2 mM denatonium benzoate (D) and various combinations of apical K2P channel blockers (bupivacaine (B), 0.2 mM) or basolateral potassium channel blockers (30 µM clofilium and 100 µM clotrimazole (C/C)). All cultures were initially treated with 10 µM amiloride to block ENaC, and at the end of the experiment with 10 µM Inh172 and 100 µM IBMX/10 µM Forskolin (I/F). **a** Cultures treated with (solid line) apical 0.2 mM bupivacaine (K2P channel blocker) have a diminished response to denatonium. **b** Basolateral potassium channel blockers pre (solid line) or post (dashed line) denatonium treatment do not affect denatonium-induced Isc. **c** Summary data demonstrating a significant (*p* < 0.0001) decrease in denatonium-benzoate induced Isc with bupivacaine pretreatment but not clofilium and clotrimazole pre-treatment. **d** Representative Ussing chamber current tracing of cultures treated with a combination of apical K2P channel blocker and basolateral potassium channel blockers. **e** Summary data demonstrating a significant (*p* = 0.0013) reduction in I/F-mediated reversal of the denatonium-induced current changes with pre-treatment with the combination of apical bupivacaine and basolateral clofilium/clotrimazole. There is a non-significant trend toward reduction of I/F-mediated current changes with apical bupivacaine or basolateral clofilium/clotrimazole pretreatment. Shown in Panel C and E are the individual data points, mean and standard error of the mean

We then tested if changes in potassium channel function and K + ion movement underlies the denatonium-mediated changes in epithelial current by assessing the influence of both apical and basal potassium channel blockers on these currents. We specifically tested bupivacaine, which blocks the human TREK family of two-pore potassium (K2P) channels [31], and the combination of clofilium and clotrimazole (C/C) to block basolateral potassium channels [32]. We found that sinonasal epithelial cultures treated with bupivacaine had more positive Isc, and then have a diminished response to denatonium benzoate (Fig. 3a, c). In contrast, basolateral potassium channel blockers did not influence denatonium-mediated changes in Isc when applied either before or after denatonium benzoate treatment (Fig. 3b, c). The IBMX/Forskolin-mediated reversal of the current changes observed with denatonium benzoate were partially reduced by apical bupivacaine (Fig. 3a) or basolateral clofilium/clotrimazole (Fig. 3c), respectively. However, this trend toward attenuation of the observed response to IBMX/Forskolin was not statistically significant (Fig. 3e). Interestingly, the combination of 0.2 mM bupivacaine to block apical K2P channels combined with 30 µM clofilium and 100 µM clotrimazole to block basolateral potassium channels treatment significantly (*p* = 0.0013) inhibited the ability of IBMX/Forskolin to reverse denatonium-induced changes in Isc (Fig. 3d, e). Taken together, these data suggest that closure of apical K2P channels is important for the denatonium-mediated changes in epithelial current, and that both apical and basolateral K^+^ channels are important in the cAMP-dependent reversal of these effects.

### Blocking potassium channels enhances respiratory epithelial antimicrobial peptide release

**Fig. 4:**
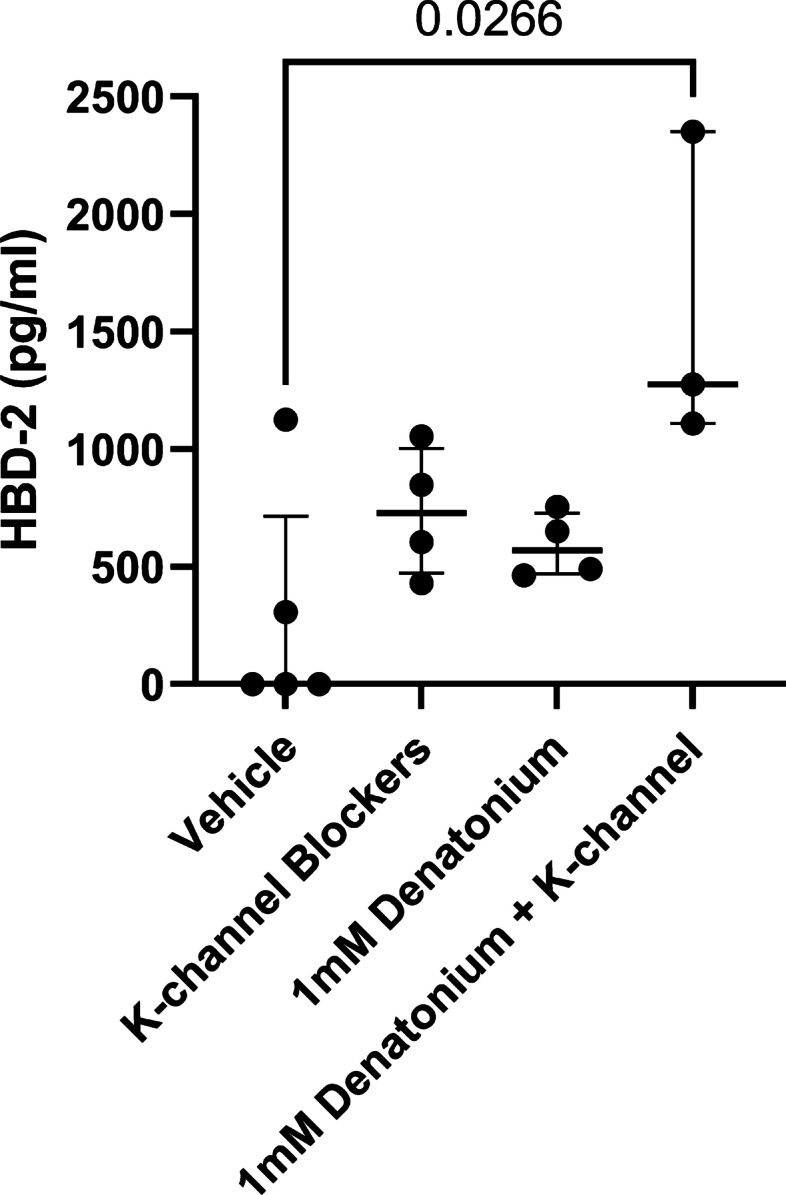
Denatonium-mediated antimicrobial peptide release is increased by blocking potassium channels. There is an increase in human β-defensin-2 (HBD-2) release from the apical surface of sinonasal ALI cultures treated with 1 mM denatonium benzoate plus K-channel blockers (apical 0.2 mM bupivacaine, basal 30 µM clofilium and 100 µM clotrimazole) compared to vehicle control (*p* = 0.00266), as well as a non-significant trend toward increased HBD-2 compared to 1 mM denatonium-benzoate alone or K-Channel blockers alone. Statistical significance determined by Kruskal–Wallis test followed Dunn’s multiple comparison with statistical significance *p* ≤ 0.05. Individual data points as well as median and interquartile range are shown

Regulation of potassium leak currents controls the resting membrane potential of a cell and regulates its excitability, where closure of potassium leak channels can lower the threshold for membrane depolarization and cell excitability [33]. Given the possible role of denatonium-benzoate regulating closure of potassium channels suggested by these data (Fig. 2), and therefore in altering resting membrane potential, we tested if these denatonium-dependent changes in potassium channel function would augment HBD-2 release by these human upper airway epithelial cells. There was an increase in HBD-2 release from sinonasal ALI cultures treated with apical bupivacaine and basal potassium channel blockers (clofilium/clotrimazole) (median [HBD-2] 728 pg/ml, interquartile range (473–1004) pg/ml), or with apical 1 mM denatonium benzoate (median [HBD-2] 570 pg/ml, interquartile range (470–729) pg/ml) compared to vehicle (control)-treated cultures (median [HBD-2] 0 pg/ml, interquartile range (0–716) pg/ml) (Fig. 4). These observed changes in HBD-2 release were not statistically significant. The combination of potassium channel blocker treatment together with apical 1 mM denatonium benzoate treatment resulted in a significant (p = 0.0266) increase in HBD-2 release (median 1275 pg/ml, interquartile range (1110–2350) pg/ml) (Fig. 4). These data suggest that closure of potassium channels lowers the threshold for stimulation of defensin release by denatonium benzoate.

## Discussion

In these investigations, our data suggests that the bitter agonist, denatonium, alters ion transport by two-pore potassium channels through a cAMP-regulated process. Potassium leak currents are regulated by the families of one and two-pore potassium (K2P) channels, which are expressed across a wide range of cell types including cardiovascular, gastrointestinal (GI) and neural cells [33, 34]. These potassium leak currents are controlled by both basal and apical potassium channels and are critical in maintaining resting membrane potential [33]. Relevant to this work, multiple subtypes of K2P channels are expressed in human bronchial epithelial cells [35]. K2P channels have roles in multiple physiologic processes, including responses to mechanical stress, taste, temperature, and pH [33, 34, 36, 37]. Furthermore, closure or blockade of these potassium leak channels can reduce membrane hyperpolarization and allow for increased cell excitability [33].

**Fig. 5:**
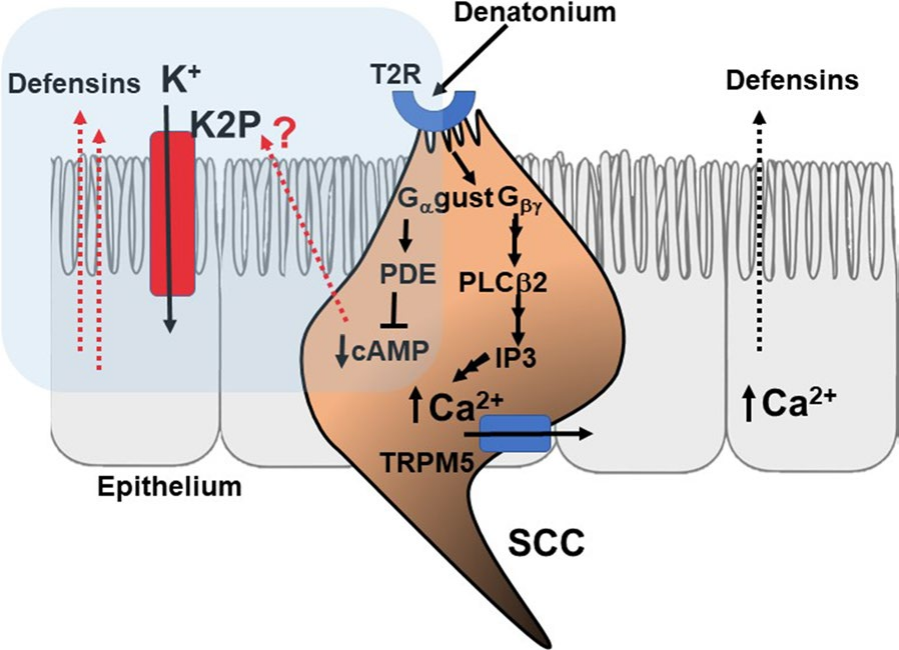
Proposed Model for bitter taste receptor stimulation of G_α_ and G_βγ_ pathways in human respiratory epithelium. Denatonium stimulation of bitter taste receptors on SCCs activates both calcium and cAMP as downstream signaling molecules that can induce epithelial defensin release. cAMP-mediated closure of apical potassium channels may reduce the threshold for calcium-mediated defensin release

Denatonium stimulation of bitter taste receptors on SCCs activates calcium-dependent taste signaling through a PLCβ-2 and IP_3_ mediated process [4]; this process ultimately results in increased β-defensin (antimicrobial peptide) release [4]. Maintenance of epithelial membrane polarity is important for organized, directional epithelial transport [38] and it is possible that reducing membrane hyperpolarization also lowers the threshold for apical protein secretion. It is therefore possible that closure of potassium channels in these experiments lowers the epithelial threshold for calcium signaling and subsequent defensin release. This suggests that bitter receptor stimulation of G_βγ_-associated calcium signaling may be amplified by G_α_-gustducin-associated cAMP signaling (Fig. 5). Further, the finding that closure of potassium channels amplifies denatonium-mediated defensin release directly implicates potassium currents in regulating respiratory epithelial defensin release. The specific mechanism(s) underlying this process in epithelial cells is/are unknown at this time but could involve additional changes in voltage gated calcium channels or activation of TRP channels that can also amplify calcium signaling.

The broad epithelial down-regulation of K2P channel function elicited by denatonium benzoate observed in these experiments also suggests that there may be a broader array of epithelial pathways affected by bitter taste receptors agonists through regulation of potassium channel function. Closure of K2P channels by bitter compounds may have a broader role in regulation of the respiratory inflammatory response, as a deficiency of TREK-1 (subtype of K2P channel) in mouse airway epithelial cells reduces interleukin-6 secretion [39]. Closure of K2P channels can also modulate sodium absorption and chloride secretion [35] and it is possible that bitter byproducts of bacterial metabolism through inhibition of K2P channels could alter mucus composition and reduce mucociliary clearance thus contributing to mucus stasis observed in acute and chronic upper respiratory bacterial infections. The data presented here also suggest that the observed denatonium benzoate-mediated current changes were at least partially due to activation of CFTR (Fig. 2a). It is possible that deficiencies in CFTR function [40], such as those observed in cystic fibrosis, could dampen bitter agonist-mediated changes in epithelial current. This could potentially contribute to pathologic changes in the mucus ion composition and may also reduce defensin release which could promote chronic bacterial colonization typically observed in those with cystic fibrosis.

The data presented here, as well as prior observations [22, 23] that cAMP serves as an additional downstream messenger of epithelial taste receptors and may also amplify the classical G_βγ_-mediated calcium signaling downstream of SCC bitter taste receptors, has parallels to taste cells where cAMP may be an additional downstream messenger. Lowering cAMP levels augments calcium signaling in taste cells [18, 19]. Our data suggesting the reversible nature of the denatonium-induced current changes by increasing intracellular cAMP (100 µM IBMX/10 µM Forskolin treatment), coupled with the blockade of this current reversal with direct potassium channel inhibition, together suggest that the effect of denatonium benzoate on potassium channel currents is mediated by cAMP rather than an off-target effect of denatonium. Human upper respiratory defensin release in response to denatonium was previously shown to be reduced in the presence of forskolin further indicating that cAMP is a downstream signal for denatonium in respiratory epithelium [4].

cAMP-mediated regulation of K2P channels has been demonstrated in other mammalian cell types. The threshold for closure of K2P channels is regulated by phosphorylation/ dephosphorylation, including by cAMP/protein kinase A- and PIP_2_/protein kinase C-dependent pathways [33, 34, 37, 41]. Prior work also supports regulation of K2P channels by other GPCRs through their respective G proteins; activation of G_αq_ or G_αs_ inhibits K2P function whereas activation of G_αi_ enhances K2P channel function [42]. The specific mechanism(s) of denatonium-mediated changes in potassium current is/are yet to be determined. Such mechanism(s) could involve changes in the phosphorylation state of potassium channels resulting in epithelial potassium channel closure [43] or internalization of potassium channels.

## Conclusions

The on-demand secretion of antimicrobial peptides from human sinonasal respiratory epithelium is stimulated, in part, by epithelial SCCs; this process involves calcium-mediated signaling [4] and is augmented by epithelial potassium channel current inhibition. Bitter-agonist regulation of potassium leak currents that are suggested by our data may reduce membrane hyperpolarization and lower the threshold for apical protein secretion. This may serve as an additional means of rapid regional epithelial signaling, and further study of these pathways may provide new insights into regulation of mucosal ionic composition and innate mechanisms of epithelial defense against infection.

## Data Availability

The datasets used during the current study are available from the corresponding author on reasonable request.
